# Loss of homeostatic functions in microglia from a murine model of Friedreich's ataxia

**DOI:** 10.1016/j.gendis.2023.101178

**Published:** 2023-11-23

**Authors:** Ilaria Della Valle, Martina Milani, Simona Rossi, Riccardo Turchi, Flavia Tortolici, Valentina Nesci, Alberto Ferri, Cristiana Valle, Daniele Lettieri-Barbato, Katia Aquilano, Mauro Cozzolino, Savina Apolloni, Nadia D'Ambrosi

**Affiliations:** a Department of Biology, University of Rome Tor Vergata, Rome 00133, Italy; b PhD Program in Cellular and Molecular Biology, University of Rome Tor Vergata, Rome 00133, Italy; c Institute of Translational Pharmacology, National Research Council (CNR), Rome 00133, Italy; d Santa Lucia Foundation IRCCS, Rome 00179, Italy; e Department of Systems Medicine, “Tor Vergata” University of Rome, Rome 00133, Italy

Friedreich's ataxia (FRDA) is a rare genetic disorder characterized by motor discoordination and cerebellar involvement due to mutations in the frataxin (FXN) gene, which encodes a mitochondrial protein involved in iron-sulfur cluster biogenesis and iron handling.^1^ While progress has been made in understanding FRDA's pathophysiology and cerebellar degeneration caused by frataxin deficiency, the role of central nervous system (CNS)-resident non-neuronal cells, as microglia, necessitates further investigation. Microglia play crucial roles in CNS development, neurogenesis, apoptosis, and synaptic remodeling, acting as sentinels with homeostatic functions. In neurodegenerative diseases, microglia respond rapidly to injury, potentially leading to sustained neuroinflammation and therefore, contributing to foster neuron damage.^2^ Similarly, in FRDA, cerebellar neuron degeneration may be influenced by glial cells in a non-cell-autonomous process. Indeed, in FRDA, microglia contribute to reactive oxygen species accumulation in the CNS, particularly in cerebellar regions, possibly participating in cerebellar susceptibility in ataxias. Circumstantial evidence supports the involvement of neuroinflammatory mechanisms in FRDA pathogenesis,^3^ as indicated by the presence of hypertrophic and reactive microglia in brain regions of FRDA mouse models and patients, with increased neuroimmune activity correlating with earlier symptom onset and shorter disease duration.^4^ However, the extent to which microglia dysfunction contributes to FRDA remains uncertain.

In this study, we performed a multi-layer characterization of microglia derived from the KIKO mouse model of FRDA and analyzed whether they contribute to neuron demise in the disease. Primary microglia were isolated specifically from the cerebellum, the most affected CNS area in FRDA. Before comparing WT and KIKO microglia phenotypes, we confirmed that the FXN mRNA was down-regulated in KIKO cells (Fig. S1A), and we demonstrated that FXN loss did not affect the overall number of microglia cells obtained from the cerebella of KIKO mice (Fig. S1B). We then analyzed if FXN depletion influences microglia morphology. We demonstrated that KIKO microglia displayed changes in cell shape and complexity (Fig. 1A), with decreased circularity and increased perimeter and Feret's diameter, in comparison to WT cells (Fig. S2A). To investigate if these differences in microglia morphology were related to abnormal functional states, we compared the ability of cerebellar microglia from WT and KIKO mice to migrate and perform phagocytosis. We observed that KIKO microglia displayed significantly lower spontaneous migration than WT cells (Fig. 1B; Fig. S2A). In addition, KIKO microglia showed an increase in the number of phagocytic cells, as well as in phagocytic activity per cell (Fig. 1C). Migration (Fig. S3A) and phagocytosis (Fig. S3B) were partially rescued by the iron chelator deferiprone in KIKO cells, suggesting that disturbed iron regulation, resulting from frataxin deficiency, could contribute to the observed changes in microglial phenotypes. Overall, these results demonstrate that primary microglia derived from the cerebellum of WT and KIKO mice are morphologically and functionally different, with the KIKO cells less motile but more phagocyting than the WT counterpart.

**Figure 1:**
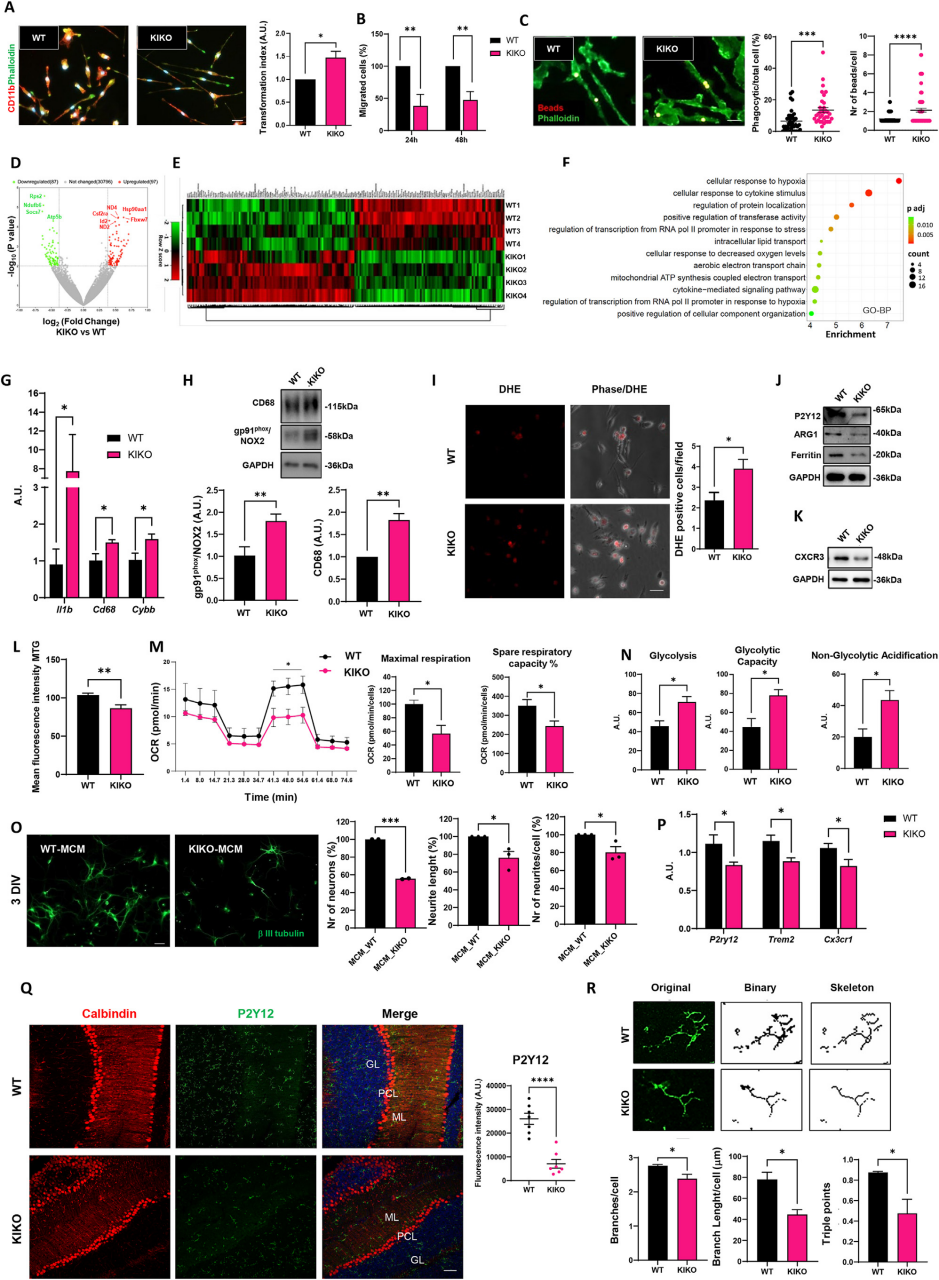
Loss of homeostatic functions in microglia from a murine model of Friedreich's ataxia. **(A)** Representative images of microglia from WT and KIKO mice stained with CD11b (red), phalloidin (green), and DAPI (blue). The transformation index is calculated as (perimeter)^2^/4π × area. Scale bar = 20 μm. The data represent mean ± standard error of the mean (SEM); *n* = 3 independent experiments. Statistical significance was calculated by *t*-test; ^∗^*P* < 0.05, ^∗∗^*P* < 0.01. **(B)** In the migration assay, the number of cells migrated into the gap was counted after 24 and 48 h and reported as a percentage of the number of WT cells. **(C)** Representative fluorescence images showing WT and KIKO microglia stained with phalloidin (green) after incubation with fluorescent latex beads (red, merged signals in yellow). The percentage of phagocytic cells/total cells and the number of beads/cells were calculated. Scale bar = 20 μm. In (B,C), the data represent mean ± SEM; *n* = 3 independent experiments, *n* = 10 fields/experiment. Statistical significance was calculated by *t*-test; ^∗∗^*P* < 0.01, ^∗∗∗^*P* < 0.001, ^∗∗∗∗^*P* < 0.0001. **(D)** The volcano plot of genes showing the magnitude (log2 (fold change/FC), *x*-axis) and significance (−log10 (*P* value), *y*-axis) for KIKO compared with WT microglia (*n* = 4/group). Differentially expressed (DE) genes are marked in red (up-regulated genes) or green (down-regulated genes). Gene names of the top 10 DE genes are shown. **(E)** Hierarchical clustering heatmap of DE genes with *P* value < 0.01 and FC > 1.3 of KIKO microglia compared with WT microglia. **(F)** Gene Ontology analysis of DE genes between KIKO and WT mice microglia. DE genes with *P* value < 0.01 and FC > 1.3 were analyzed by the Enrichr analysis tool. The top 12 enriched terms for Gene Ontology analysis are displayed based on decreasing −log10 (*P* value). BP, biological process. **(G)** Real time PCR for *Il1B*, *Cd68*, and *Cybb* in WT and KIKO microglia. The data were normalized to *Actb* and expressed as mean ± SEM of 3 (*n*) experiments performed in triplicate. **(H)** Representative western blots and quantification of CD68 and gp91^phox^ in WT and KIKO microglia. GAPDH was used as a loading control. **(I)** Representative images of dihydroethidium (DHE)-stained WT and KIKO microglia and relative DHE positive cell quantification. Scale bar = 20 μm. Representative western blots and quantification of P2Y12, arginase 1 (ARG1), ferritin **(J)**, and C-X-C chemokine receptor type 3 (CXCR3) **(K)** in WT and KIKO microglia. GAPDH was used as a loading control. In (H–K), the values were expressed as mean ± SEM; *n* = 3 independent experiments. Statistical significance was calculated by *t*-test; ^∗^*P* < 0.05, ^∗∗^*P* < 0.01, ^∗∗∗∗^*P* < 0.0001. **(L)** Quantification of the flow cytometry analysis of mitochondrial mass, using the Mito Tracker Green (MTG) fluorescent dye. **(M)** Measurement of the rate of oxygen consumption rate (OCR) in WT and KIKO microglia. Individual parameters for maximal respiration and spare respiratory capacity are indicated. **(N)** Glycolysis, glycolytic capacity, and non-glycolytic acidification were analyzed in WT and KIKO microglia. In (L–N), the data were presented as mean ± SEM; *n* = 4 independent experiments. Statistical significance was calculated by *t*-test; ^∗^*P* < 0.05. **(O)** Representative images of neurons grown in microglial conditioned medium derived from WT microglia (WT_MCM) and from KIKO microglia (KIKO_MCM), after labeling neurons with anti-β-tubulin III (green). Cell survival and length and number of neurites per cell were analyzed using the ImageJ software NeuronJ plug-in. The data represent mean ± SEM of 3 (*n*) independent experiments performed in quadruplicate. Statistical significance was calculated by *t*-test; ^∗∗∗^*P* < 0.001, ^∗^*P* < 0.05. **(P)** Real time PCR for *P2ry12*, *Trem2*, and C*x3cr*1 mRNA in the cerebellum of WT and KIKO mice at postnatal day 15. The data were normalized to *Actb* and expressed as mean ± SEM of 3 (*n*) independent experiments performed in triplicate. **(Q)** Representative confocal images of cerebellar sections from WT and KIKO mice at postnatal day 15 immunostained for P2Y12 (green) and calbindin (red). DAPI was used for nuclei staining. Scale bar = 100 μm. GL, granular layer; ML, molecular layer; PCL, Purkinje cell layer. P2Y12 signal quantification was performed by ImageJ software. **(R)** Example of original, binary, and skeletonized microglia. Analysis of the microglial branches, average branch length, and triple points of microglia (junctions with exactly three branches). In (Q,R), the values were expressed as mean ± SEM; *n* = 3 mice/group; at least 3 sections for mice. Statistical significance was calculated by *t*-test; ^∗^*P* < 0.05.

To gain insights into the overall gene expression signature of KIKO versus WT microglia and identify genes that can be relevant to the different phenotypes described, we performed transcriptomics profiling to investigate differentially expressed genes and pathways. A total of 184 genes were significantly (*p* < 0.01) differentially expressed between WT and KIKO microglia, with absolute fold-change of 1.3 or greater, as indicated on the volcano plot and heatmap, and among these, 87 genes were down-regulated and 97 up-regulated (Fig. 1D, E and Table S1). The top 10 differentially expressed genes by *p*-value encompass genes involved in mitochondria oxidative phosphorylation (*Ndufb6*, *ND2*, *ND4*, and *Atp5b*), inflammation (*Socs*, *Csf2ra*), and proteostasis (*Rps2*, *Hsp90aa1*, and *Fbxw7*) as shown in the volcano plot (Fig. 1D). Reactome pathway (Fig. S4A) and Gene Ontology (GO) (Fig. 1F; Fig. S4B) enrichment analysis indicated that differentially expressed genes were related to categories as “cellular response to cytokine stimulus”, “signaling by interleukins”, “cytokine signaling”, “immune system”, “mitochondrial membrane”, “mitochondria respiratory chain complex I”, “aerobic electron transport chain”, “NADH oxidoreductase activity”, and “positive regulation of cellular component organization”, highlighting that KIKO microglia exhibit altered morphology, proinflammatory features, and dysfunctional mitochondria.

To investigate if the differences in proinflammatory features obtained by transcriptomic analysis correlate to specific inflammatory gene signatures, we analyzed in WT and KIKO microglia the expression of genes involved in pro- and anti-inflammatory functions. As shown in Figure 1G, pro-inflammatory genes such as *interleukin-1 beta* (*IL1B)*, *Cd68*, and *NADPH oxidase* 2 *(Cybb)* are significantly up-regulated in KIKO microglia at the level of mRNA expression. The CD68 protein content is accordingly increased in KIKO cells (Fig. 1H). Remarkably, the expression of the transmembrane subunit of NOX2, gp91phox, is significantly up-regulated in KIKO microglia (Fig. 1H), suggesting that cytosolic reactive oxygen species production is increased in FXN-depleted cells. Consistently, by measuring the oxidation of the fluorescent probe dihydroethidium we verified that superoxide levels are augmented in FRDA microglia with respect to WT cells (Fig. 1I). Since it has been reported that intracellular reactive oxygen species act as second messengers to regulate downstream inflammatory pathways, we further demonstrated that nuclear factor-kappa B, the master regulator of pro-inflammatory signaling, is increased in KIKO microglia (Fig. S5A). In parallel, we observed that the protein level of genes characterizing microglial homeostatic phenotypes, such as P2Y12, arginase 1, and ferritin, was decreased in KIKO microglia (Fig. 1J; Fig. S5B). Finally, the expression of the protein C-X-C chemokine receptor type 3, related to the recruitment and function of microglia, is decreased in KIKO cells (Fig. 1K; Fig. S4C), suggesting its participation in the altered migratory and phagocytic activity found in these cells.

Since the transcriptomics results indicated alterations in mitochondria from KIKO microglia, we sought to evaluate this aspect. Flow cytometry analysis showed that in KIKO microglia, the overall mass of mitochondria labeled with the MitoTracker green fluorescent probe decreased with respect to WT cells (Fig. 1L). When we examined the oxygen consumption rates of WT and KIKO microglia, we observed defects in mitochondrial respiration (Fig. 1M). Indeed, although basal respiration and ATP production were not significantly affected in KIKO microglia (data not shown), maximal respiration and spare respiratory capacity were decreased (Fig. 1M). On the other hand, we demonstrated that KIKO microglia up-regulated overall glycolytic function, by increasing glycolysis and glycolytic capacity (Fig. 1N), suggesting a metabolic altered microglia profile in this pathological context. Overall, these results suggest that the lack of FXN impairs mitochondria functionality, with decreased oxygen consumption, enhanced glycolysis, and oxidative stress, together with increased phagocytic activity. These alterations are typical of reactive microglia and are generally associated with a cytotoxic function.^5^

To examine the possible functional effects of FRDA microglia in microglia–neuron interactions, we cultured WT mouse cortical neurons in the presence of microglial conditioned medium from WT and KIKO microglia. Immunofluorescence analysis showed that the microglial conditioned medium derived from KIKO microglia decreased neurite length, neurite number per cell, and overall neuron viability (Fig. 1O). These results provide evidence that microglia can participate in neuron degeneration in FRDA.

It has been demonstrated that KIKO mice show cerebellar synaptic deficits and dysregulated circuits at asymptomatic age.^6^ Since microglia participate in numerous developmental events in the CNS, with the aim of highlighting possible microglial dysfunctions *in vivo* during an early developmental phase of the disease, we analyzed the cerebellum of WT and KIKO mice at postnatal day 15. As expected, FXN levels were significantly reduced in cerebellar homogenates (Fig. S6A) and sections (Fig. S6B) of KIKO mice compared with age-matched controls. We demonstrated that the mRNA levels of *P2ry12*, as well as of *triggering receptor expressed on myeloid cells 2 (Trem2)* and *chemokine (C-X3-C motif) receptor 1* (C*x3cr*1), key microglial molecules mediating the process of synapse refinement during neurodevelopment, were decreased in the cerebellum of KIKO mice (Fig. 1P). Importantly, the P2Y12 protein, which characterizes cells in a homeostatic context, decreased in molecular, Purkinje cell and granular cerebellar layers of KIKO mice (Fig. 1Q). Finally, consistently with the *in vitro* results, we demonstrated changes in microglia morphology in the cerebellum of WT and KIKO mice. We found a significant reduction in the number of branches per cell, in the branch length, and in the triple endpoints in KIKO microglia, which became overall less ramified compared with WT cells (Fig. 1R), indicating that their ability to generate complex branches is impaired in KIKO cerebellum.

Although several reports correlated microglia morphology to cerebellar degeneration in FRDA *in vivo,* this work is the first to provide multilayer evidence (phenomics, transcriptomics, and metabolic analysis) that FRDA microglia are dysfunctional, suggesting a contribution of non-cell autonomous mechanisms in FRDA pathogenesis. Considering the established role of microglia in neurodegeneration, the comprehension of the mechanisms underlying microglial-related pathological mechanisms in FRDA could be instrumental in designing time- and molecule-targeted therapeutic interventions to halt cerebellar degeneration in the disease.

## Ethics declaration

All animal experiments followed the European Guidelines for the use of animals in research (2010/63/EU), the Italian Laws (D.L. 26/2014), and the national (Ministry of Health, licenses n° 324/218-PR and n° 210/202-PR) committees, in compliance with FELASA Recommendations.

## Author contributions

Savina Apolloni and Nadia D'Ambrosi contributed to the study's conception and design. Material preparation and data collection and analysis were performed by Ilaria Della Valle, Martina Milani, Simona Rossi, Riccardo Turchi, Flavia Tortolici, Valentina Nesci, Cristiana Valle, Alberto Ferri, Daniele Lettieri-Barbato, Katia Aquilano, and Mauro Cozzolino. The first draft of the manuscript was written by Savina Apolloni and Nadia D'Ambrosi. All authors commented on previous versions of the manuscript. All authors read and approved the final manuscript.

## Funding

This work was funded by the 10.13039/100002243National Ataxia Foundation (NAF) and 10.13039/100002108Friedreich's Ataxia Research Alliance (10.13039/100002108FARA) (*n*° 821396 [RG]) to Nadia D'Ambrosi, by Next Generation EU PRIN PNRR 2022 (N° P2022JKTWH) to Nadia D'Ambrosi and Mauro Cozzolino, by 10.13039/100002108FARA Research Grant 2019 to Katia Aquilano, and by 10.13039/100002108FARA Research Grant 2021 to Daniele Lettieri-Barbato.

## Data availability

The datasets generated and analyzed during the current study are available in the Gene Expression Omnibus (GEO) repository, under the accession number GSE232826.

## Conflict of interests

The authors declare no competing financial interests.
