# Supplementary material for: Loss of homeostatic functions in microglia from a murine model of Friedreich's ataxia
Source: Genes Dis. 2023 Nov 23;11(6):101178. doi: 10.1016/j.gendis.2023.101178 (PMC11295442; doi:10.1016/j.gendis.2023.101178)
Supplement: Multimedia component 1 [file mmc1.docx]

**Materials and methods**

**KIKO mice**

Procedures involving animal care were conducted at Tor Vergata University Animal Facility (CIMETA) in compliance with FELASA Recommendations, European Guidelines for the use of animals in research (2010/63/EU), Italian Laws (D.L. 26/2014) and national (Ministry of Health, license no. 324/2018-PR) committees. Adult B6.Cg-Fxntm1MknFxntm1Pand/J (Stock No: 014162) (Knock-in knock-out, KIKO) mice, where microglia reactivity was previously demonstrated, were obtained from Jackson Laboratories (Maine USA). KIKO mice are generated by animals bearing a (GAA)_230_ expansion repeat “knock in” targeted to the endogenous *FXN* locus coupled with a *FXN* targeted “knock out” mutation allele disrupting exon 4. Littermate C57BL/6 mice wild-type (WT) were used as controls. Mice were maintained at 21.0 °C and 55.0 ± 5.0% relative humidity under a 12 h/12 h light/dark cycle. Food and water were given *ad libitum*.

**Antibodies for western blot (WB) and immunofluorescence (IF)**

| **Antibody** | **Host** | **Application** | **Diluition** | **Company** |
| --- | --- | --- | --- | --- |
| ARGINASE 1 | Rabbit | WB | 1:700 | Abcam |
| CD68 | Rat | WB | 1:500 | AbD Serotec |
| CXCR3 | Rabbit | WB  IF | 1:500  1:200 | Bioss Antibodies |
| GAPDH | Mouse | WB | 1:2500 | Calbiochem |
| NOX2 /gp91^phox^ | Mouse | WB | 1:1000 | BD Transduction Laboratories |
| FERRITIN | Rabbit | WB | 1:1000 | Abclonal |
| _NF-кB_ | Rabbit | WB | 1:500 | CST |
| P2Y12 | Rabbit | WB  IF | 1:200  1:200 | Anaspec |
| CD11b | Rat | IF | 1:200 | BioRad |
| βIII-TUBULIN | Rabbit | IF | 1:500 | Cell Signaling |
| CALBINDIN | Mouse | IF | 1:200 | Sigma |
| FXN | Rabbit | WB  IF | 1:1000  1:200 | Santa Cruz |
| Anti-IgG-HRP conjugated | Rabbit | WB | 1:2500 | Jackson ImmunoResearch |
| Anti-IgG-HRP conjugated | Mouse | WB | 1:5000 | Jackson ImmunoResearch |
| Anti-IgG-HRP conjugated | Rat | WB | 1:2500 | Jackson ImmunoResearch |
| Anti-IgG-Alexa 488 | Rabbit | IF | 1:200 | Jackson ImmunoResearch |
| Anti-IgG-Cy3 | Mouse | IF | 1:200 | Jackson ImmunoResearch |
| Anti-IgG-Alexa 488 | Rat | IF | 1:200 | Jackson ImmunoResearch |

**Primary microglia cell cultures**

Primary microglia cultures from cerebellum were prepared as previously described. Briefly, 5–6-day-old (p6) mice were sacrificed and, after removing the meninges, the cerebellum was minced and digested with 0.01% trypsin and 10 μg/ml DNaseI. Following the process of dissociation and filtration through 70 µm filters, the cells were re-suspended and placed in DMEM/F-12 media with GlutaMAX™ (Gibco, Invitrogen, UK). This media was supplemented with 10% fetal bovine serum (FBS), 100 Units/ml of gentamicin, and 100 µg/ml of streptomycin/penicillin. The cells were then plated at a density of 62,500 cells/cm^2^. After approximately 15 days, a mild trypsinization in DMEM/F-12 without FBS (0.08% trypsin in DMEM/F-12 without FBS) was performed for 40 min at 37°C to remove non-microglial cells [18]. The resultant adherent microglial cells (>98% pure) were cultured in 50% mixed glial cells conditioned medium at 37°C in a 5% CO2 and 95% air atmosphere for 48 h until use.

**Microglia morphological analysis**

For morphological analysis, microglia cultures were stained with CD11b and images were acquired by Zeiss fluorescence microscope. For each culture, about 100 random microscope fields per well from at least 20 well were counted and analyzed using ImageJ (NIH, USA). For the measurement of area (A, in μm^2^), perimeter (P, in μm) and transformation index (TI), CD11b-positive microglial images were converted into binary replicas using thresholding procedures implemented by the ImageJ. The TI, which is a measure of differentiated cell morphology, was determined according to Fujita et al. (1996), using the following formula: [perimeter of the cell (μm)]^2^/4π [cell area (µm^2^)].

**Migration assay**

For the migration assay, 40,000 microglia cells were seeded in mini chamber inserts (Ibidi, USA). Deferiprone (DFP) was added at 150 μM. After 24 hours, the inserts were removed, and the cells were analyzed. The bright-field images of the migration assay were photographed at 20X magnification after 0, 24 and 48 hours. Cell motility was determined by counting the number of cells that migrated toward the wound. Each experiment was carried out in triplicate.

**Phagocytosis assay**

Microglia cells were cultured into 24-well at appropriate densities to ensure the cells were confluent to approximately 70,000 per well at the time of assay. Deferiprone (DFP) was added at 150 μM. Fluorescent red latex beads (2 μm diameter, Sigma) pre-opsonized in 50% FBS were loaded to the cells at a concentration of 1x10^6^ beads/ml and kept at 37°C for 3 h. After that, the cells underwent a washing step using phosphate-buffered saline (PBS) to eliminate any non-phagocytosed beads. Subsequently, they were fixed in a solution containing 4% paraformaldehyde (PFA). After phalloidin and 4',6-diamidino-2-phenylindole (DAPI) counterstaining the number of beads ingested by the cells was determined using a Zeiss fluorescence microscope. For each well, DAPI and phalloidin images were collected (n=15–20) and analysis was done by ImageJ software.

**Immunofluorescence and confocal microscopy**

Primary cells were fixed for 15 minutes in 4% paraformaldehyde, and permeabilized for 5 minutes in PBS containing 0.1% Triton X-100. The cells were kept for 2.5 hours at 37°C with the appropriate primary antibody and then stained for 1 hour with the appropriate secondary antibody. Phalloidin (1:200, Sigma Aldrich, Italy) was used to stain cell actin filaments. For nuclear staining, the cells were exposed to a 1 μg/ml concentration of 6-diamidino-2-phenylindole (DAPI) for 5 minutes. Following this, the cells were mounted and cover-slipped using Fluoromount mounting medium (Sigma).

Cerebellum slices were obtained from WT (n=3) and KIKO (n=3) mice sacrificed by cervical dislocation. Cerebella were post-fixed overnight in 4% PFA and then cryoprotected in 30% sucrose in 0.1M PBS at 4°C. Prior to cryosection, tissues were frozen at -80°C and sagittal sections of 25 μm thickness were cut on a cryostat (CM 1850, Leica, Germany). Immunofluorescence analysis was performed in free-floating according to the following procedure: sections were washed in PBS and blocked in PBS containing 10% normal donkey serum and 0.3% Triton X-100 for 1 hour at room temperature. Sections were incubated with the appropriate antibodies in PBS, 2% normal donkey serum and 0.3% Triton X-100 for 48 hours at 4°C. Sections were incubated with appropriate fluorescent-conjugated secondary antibodies in PBS, 2% normal donkey serum and 0.3% Triton X-100 for 3 hours at room temperature. After PBS washes, sections were incubated with DAPI for 5 minutes at room temperature. The slides were cover-slipped with Fluoromount mounting medium (Sigma). Immunofluorescence was analyzed by means of a confocal laser scanning microscope (LSM 510 META, Zeiss) equipped with three lasers: Argon/2, HeNe543 and HeNe633. The digital images' brightness and contrast were modified using the LSM Image Browser software (Zeiss).

**Protein extraction and western blot**

To obtain total-protein extracts from cellular cultures, the cells were collected and resuspended in ice-cold RIPA buffer which consisted of PBS, 1% Nonidet P-40, 0.5% sodium deoxycholate, and 0.1% SDS. Tissues were lysed in homogenization buffer (50 mM Tris HCl pH 7.4, 250 mM NaCl, 1 mM EDTA, 5 mM MgCl_2_, 1% Triton X-100, 0.25% Na-deoxycholate, 0.1% SDS). Additionally, a protease inhibitor cocktail (Cell Signaling) was added to prevent protein degradation. The lysates were then kept on ice for 30 minutes and subsequently centrifuged at 14,000 × g for 10 minutes at 4°C. The resulting supernatants containing the protein extracts were collected and quantified using the Bradford protein assay from Bio-Rad Laboratories. Proteins were run on SDS-PAGE and transferred onto nitrocellulose membranes (GE Healthcare). The nitrocellulose membranes were blocked using 5% non-fat dry milk solution. Subsequently, the membranes were kept overnight at 4°C with the specified primary antibodies. After rinsing with Tris-buffered saline-Tween 20 (TBS-T) solution, the membranes were incubated with the suitable peroxidase-conjugated secondary antibody, which was diluted in TBS-T containing 1% non-fat dry milk. Following another round of washing, the membranes were developed using either the Enhanced chemiluminescence (ECL) detection system from Roche or the Advance Western blot detection kit from Amersham Biosciences, USA. Densitometric analysis of the resulting bands was carried out using the ImageJ software program from NIH, USA.

**Quantitative real-time PCR**

For quantitative Real-Time PCR (RT-PCR), RNAs were isolated using TRIzol (Thermo Fisher Scientific). RNAs were quantified and reverse-transcribed with random primers by GoScript Reverse Transcription System (Promega). RT-PCR was performed with GoTaq PCR Green Master Mix (Promega) according to the manufacturer’s instruction.

**Primers used in RTqPCR**

| **NAME** | **FORWARD SEQUENCE** | **REVERSE SEQUENCE** |
| --- | --- | --- |
| *Cd68* | GACCGCTTATAGCCCAAGGAA | CATCGTGAAGGATGGCAGGA |
| *Cybb* | TGAATGCCAGAGTCGGGATTT | CCCCCTTCAGGGTTCTTGATTT |
| *Il1β* | GCAACTGTTCCTGAACTCAACT | ATCTTTTGGGGTCCGTCAACT |
| *Cx3cr1* | CAAGCTCACGACTGCCTTCT | TGTCCGGTTGTTCATGGAGTT |
| *P2ry12* | TTCCCGTATCCAGGGTCACA | GGCAGCCTTGAGTGTTTCTG |
| *Trem2* | TGCTGGCAAAGGAAAGGTG | GTTGAGGGCTTGGGACAG |
| *Fxn* | TCTCTTTTGGGGATGGCGTG | GCTTGTTTGGGGTCTGCTTG |
| *Actb* | CTAAGGCCAACCGTGAAAAG | ACCAGAGGCATACAGGGACA |

**Detection of intracellular ROS levels**

Intracellular superoxide anion production was measured using Dihydroethidium (DHE, Santa Cruz Biotechnology, USA) observed under fluorescence microscope. To detect intracellular ROS levels, 70.000 primary microglia cells were seeded in a 24-well plate. After 48 hours a solution containing 10 ng/μL of dihydroethidium (DHE) was added to each well for 10 minutes in the dark. Cells were directly observed using a fluorescence microscope (Zeiss). Ten random images were captured from each well under a 20× magnification. This process was performed in triplicate for each experimental group. The fluorescence intensity of the captured images was quantified using the ImageJ software.

**RNA-seq**

Total RNAs from microglia cells were extracted using Direct-zolTM RNA MiniPrep (ZYMO RESEARCH) according to the manufacturer’s instructions. Total RNA was quantified using the Qubit 4.0 fluorimetric Assay (Thermo Fisher Scientific). Libraries were prepared from 125 ng of total RNA using the NEGEDIA Digital mRNA-seq research grade sequencing service (Next Generation Diagnostic srl) (Xiong et al., 2017) which included library preparation, quality assessment and sequencing on a NovaSeq 6000 sequencing system using a single-end, 100 cycle strategy (Illumina Inc.). The raw data were analyzed by Next Generation Diagnostic srl proprietary NEGEDIA Digital mRNA-seq pipeline (v2.0) which involves a cleaning step by quality filtering and trimming with bbduk, alignment to the reference genome (mm10) using STAR 2.6.0a, and counting by gene with HTseq-counts 0.9.1. The raw expression data were normalized and analyzed by Rosalind HyperScale architecture 2 (OnRamp BioInformatics, Inc.). The bidirectional hierarchical clustering heatmap was generated using FunRich software (version 3.1.3). Functional enrichment analysis were performed by Enrichr webtool. The resulting data was visualized using a free online platform for data analysis and visualization available at <https://www.bioinformatics.com.cn/en>.

**Analysis of mitochondrial mass**

To evaluate mitochondrial mass, primary microglia cells were incubated with 250 nM MitoTracker Green (M7514, ThermoFisher Scientific), for 15 minutes at 37°C. After washing, cytofluorimetric analysis of samples was performed by cytofluorimetry. Flow cytometry analyses were performed using Amnis CellStream Flow Cytometer (Luminex Corporation, USA) and analyzed using FlowJo software version 4.14.

**Seahorse analysis**

Bioenergetic evaluation of WT and KIKO microglia was performed by Seahorse XFe96 extracellular flux analyzer (Seahorse Bioscience-Agilent, USA) that enables reproducible measurement of oxygen consumption (OCR, an indicator of mitochondrial respiration) and extracellular acidification rate (ECAR, largely the result of glycolysis). Analyses were performed through the use of the Mitochondrial stress test kit and the Glycolysis stress test kit. All assays were conducted following the manufacturer's protocol and outlined briefly below.

WT and KIKO microglia cells were seeded (4 × 10^4^ cells/well) on micro-lysine-coated plates and incubated overnight in 5% CO_2_, 37 °C. To perform Mitochondrial Stress Test, medium was replaced with XF Base medium supplemented with 1 mM pyruvate, 2 mM glutamine, 10 mM glucose; for the Glycolysis Stress Test, the culture medium was replaced with XF Base medium containing 2 mM glutamine. The cells were then placed in a CO_2_-free incubator for 45 minutes. Subsequently, the cells were transferred to the Seahorse XFe96 analyzer for further analysis. OCR and ECAR parameters were measured after sequential drug injections (1μM oligomycin, 1.5μM FCCP, 0.5μM rotenone/antimycin A for Mitochondrial stress test and 10mM glucose, 1μM oligomycin, 50mM 2D-glucose for Glycolysis stress test).

Data were analysed with Wave software (available on Seahorse Bioscience website) and results were normalized to cell number. Representative results of a single experiment with n=10 biological replicates are shown.

**Primary neuronal cultures**

Primary cortical neurons were prepared from postnatal C57BL/6 mice (P0-P1), as described previously. Briefly, mice were decapitated, and the meninges were stripped off from the exposed brain. The cortices were removed and digested in 0.25% trypsin (Life Technologies, Spain) containing 1 mg/ml DNaseI (Sigma Aldrich) for 20 minutes at 37 °C, and then dissociated by trituration with a fire-polished Pasteur pipette. The dissociated cortical cells were plated in 6-well plate previously coated with poly-L-lysine (PLL) (1 mg/mL) or on PLL-coated coverslips and maintained in Neurobasal® medium (Gibco Life Technologies) supplemented with B-27® (Life Technologies) at densities ranging from 40,000 to 60,000 cells/cm^2^. To analyze the possible effects of conditioned medium from KIKO microglia (MCM) on neuronal growth and maturation, the medium was changed after 3 hours to MCM derived from WT or KIKO microglia.

**Neuron quantitative analysis**

For the quantitative analysis of neurite outgrowth and branching in primary neuronal cultures, the cells were immunostained with βIII-tubulin and observed using a Zeiss Axiovert 200 inverted fluorescence microscope that was equipped with a CCD camera. At least five randomly selected fields were captured for each experimental condition. The axons and dendrites were traced from the cell body to the end of their extensions, and the total distance per field was calculated using the ImageJ plugin NeuronJ. To determine the number of neurons, the total count of DAPI-positive cells was performed for each field and experimental condition.

**Skeleton analysis**

Immunofluorescence images of post-natal day 18 cerebella slices from WT and KIKO mice were captured with a confocal microscope using a 20 × objective. The characterization of microglial morphology was performed using ImageJ analysis software. Images were first pre-processed using a macroscript that applied a threshold, followed by conversion to binary images. The "Analyze Skeleton" plugin was then executed on the binarized images to analyze microglia branch number (cell process and endpoints per cell), branch length and branch junctions (triple or quadruple junctions). These data measure microglial morphology (complexity and process length). At least 50 cells per individual in each subgroup were analyzed.

**Statistical analysis**

The data represent the mean ± standard error of the mean (S.E.M). Analysis was accomplished with the statistical software package Prism v9 (GraphPad Software) using an independent t-test for differences between two groups. *p<0.05 was considered significant.

**A**

B

**Supplementary Figure 1**

**Supp. Fig. 1 FXN expression is decreased in KIKO mice but doesn’t affect microglia yield.** Microglia from the cerebellum of p6 WT and KIKO mice were obtained from mixed glial culture. (A) Real-time PCR for FXN in WT and KIKO microglia. (B) Counts of microglia cells obtained from WT and KIKO cerebellum. Data represent mean ± S.E.M. of n=3 (A) and n=16 (B) independent experiments. Statistical significance was calculated by t-test. **p<0.01

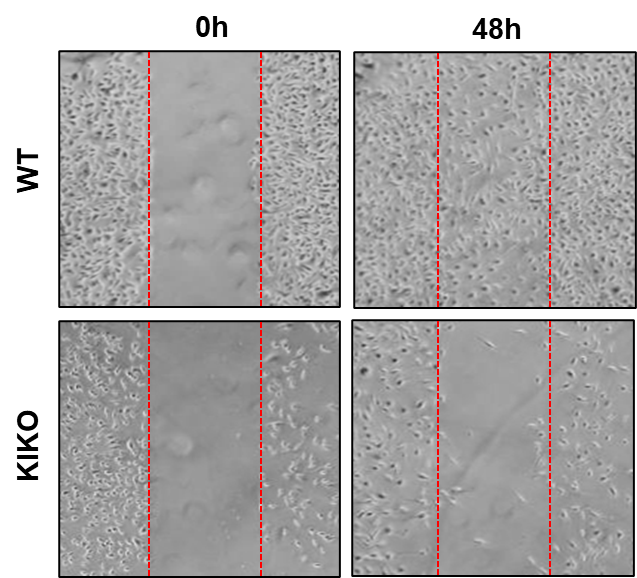


**Supplementary Figure 2**

**A**

**B**

**Suppl Fig. 2 KIKO microglia display altered cell morphology and decreased migration.** Microglia from WT and KIKO mice were analyzed by ImageJ software for different size descriptors (A). Data represent mean ± S.E.M. (n = 3 independent experiments). Statistical significance was calculated by t-test. *p<0.05, **p<0.01. (B) Primary microglia were seeded into removable mini chambers and, upon removal of the inserts, were left free to migrate. Representative micrographs show the migration assay at 48 hours from the migration start. Scale bar=100μm.

**Supplementary Figure 3**

**B**

**A**


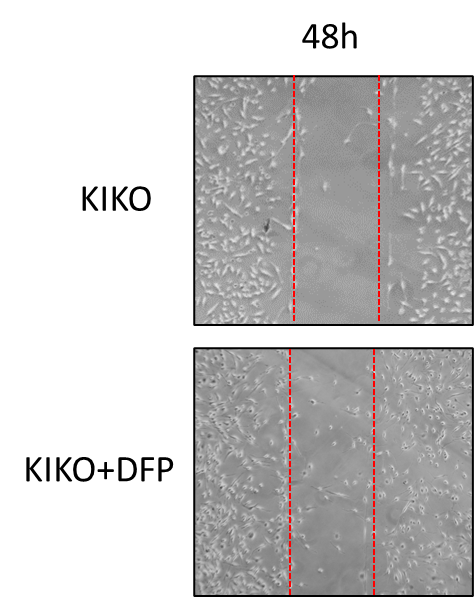

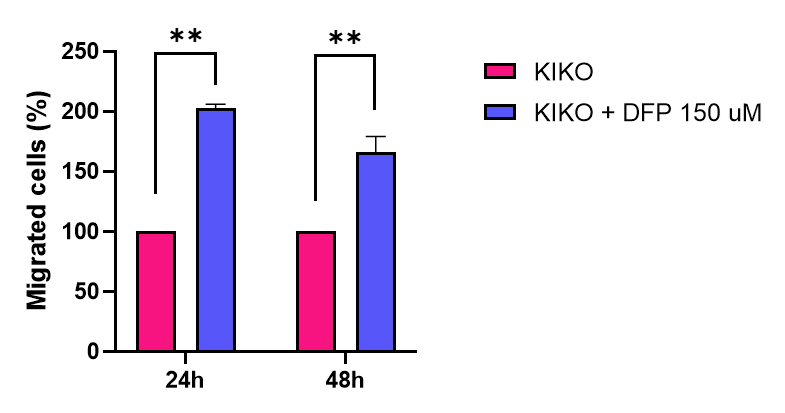

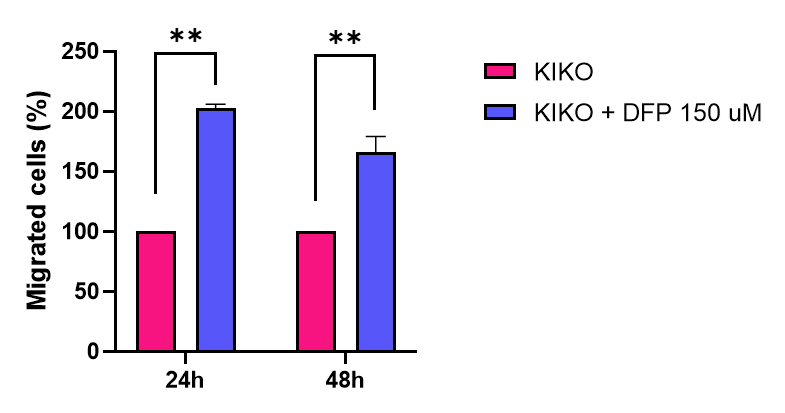


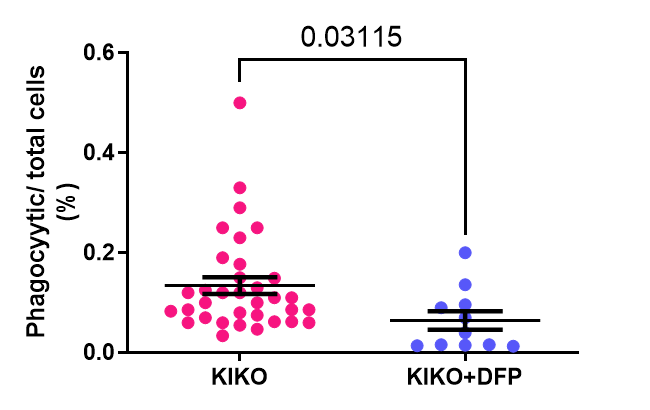


*

**Suppl Fig. 3 Iron chelation with deferiprone (DFP) affects migration and phagocytosis in KIKO microglia.** (A) Primary KIKO microglia were seeded into removable mini chambers and in the absence (KIKO) or presence of 150 μM DFP (KIKO+DFP). Representative micrographs show the migration assay at 48 h from the migration start. Scale bar=100μm. After 24 and 48 h from the removal of inserts the number of cells into the gap was counted and reported as % of the number of KIKO cells. (B) Primary KIKO microglia were assayed without (KIKO) or with 150 μM DFP (KIKO+DFP) for their phagocytic activity. The % of phagocytic cells/total cells was calculated. Data represent mean ± S.E.M. (n = 3 independent experiments). Statistical significance was calculated by t-test. *p<0.05, **p<0.01

GO-CC

GO-MF

**Supplementary Figure 4**

**A**

**B**

**Suppl Fig. 4 KIKO microglia display differential gene expression from WT cells.** Pathway analysis (A) and Gene Ontology analysis (B) of DE genes between KIKO and WT mice microglia. DE genes with P value < 0.01 and FC > 1.3 have been analyzed by the Enrichr analysis tool. Top 10 enriched terms for Reactome pathway analysis and Gene Ontology are displayed based on decreasing -log10(p value). The color code shows the adjusted p value (p adj), while the size of bubble represents the number of genes enriching the corresponding annotation (count). CC, cellular component; MF, molecular function

**Supplementary Figure 5**

**A**


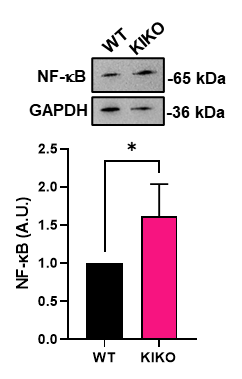


**C**

**B**

**Suppl** **Fig. 5 KIKO microglia display a pro-inflammatory gene signature.** Representative blot and quantification of NF-κB (A), and quantification of P2Y12, ARG1 and ferritin (B), and CXCR3 (C) in WT and KIKO microglia. GAPDH was used as a loading control. Values are expressed as mean ± S.E.M., n=3 independent experiments. Statistical significance was calculated by t-test ∗p<0.05, ∗∗p<0.01, ****p<0.0001

**Supplementary Figure 6**


**A B**

**Supp. Fig. 6 FXN expression is decreased in the cerebellum of KIKO mice.** (A) Representative western blot and quantification of FXN in WT and KIKO cerebellum lysates. GAPDH was used as a

loading control. Values are expressed as mean ± S.E.M., n=4 mice/group. Statistical significance was calculated by t-test ∗∗∗∗p<0.001. (B) Representative confocal images of cerebellar sections from WT and KIKO mice at postnatal day 15, immunostained for FXN (green). Scale bar: 50 μm. GL, granular layer; ML, molecular layer; PCL, Purkinje cell layer.
